# An Irish cocktail of flatworm, earthworm and parasite DNAs: genomics of invasive land flatworms (Geoplanidae) reveal infestations by two new *Mitosporidium* species (Microsporidia)

**DOI:** 10.1051/parasite/2025060

**Published:** 2025-10-17

**Authors:** Romain Gastineau, Archie K. Murchie, Leigh Winsor, Jean-Lou Justine

**Affiliations:** 1 Institute of Marine and Environmental Sciences, University of Szczecin Szczecin Poland; 2 Sustainable Agri-Food Sciences Division, Agri-Food and Biosciences Institute Belfast BT9 5PX Northern Ireland United Kingdom; 3 College of Science and Engineering, James Cook University Townsville Qld 4811 Australia; 4 ISYEB, Institut de Systématique, Évolution, Biodiversité (UMR7205 CNRS, EPHE, MNHN, UPMC, Université des Antilles), Muséum National d’Histoire Naturelle CP 51 55 rue Buffon 75231 Paris Cedex 05 France

**Keywords:** Microsporidia, Rozellomycota, Invasive Alien Species, Geoplanidae, Mitogenome

## Abstract

According to the classical Enemy Release Hypothesis, one reason for the success of invasive species is that they have escaped their predators and parasites during migration to newly invaded territories. In this context, the discovery of any parasite of an invasive species is of particular interest. Here, we report the results of genomic investigations performed on two invasive species of land flatworms (Geoplanidae) collected in Northern Ireland, *Kontikia andersoni* Jones, 1981, and *Australoplana sanguinea* (Moseley, 1877). We describe the mitogenomes and paralogous RNA genes of both species. Prey DNA was detected in both flatworm species, providing molecular evidence that their diet includes earthworms. Unexpectedly, we detected sequences assigned to the microsporidian genus *Mitosporidium* Haag *et al.*, 2015, which, prior to this study, included a single species. Each land flatworm species harboured its own species of *Mitosporidium*. For nomenclatural reasons, we could not assign binomial names to these species; instead, we designate them as *Mitosporidium* sp. JL467 (in *K. andersoni*) and *Mitosporidium* sp. JL472 (in *A. sanguinea*). For each new *Mitosporidium* species, we describe the gene content of the mitogenome and the complete cluster of nuclear ribosomal RNA genes. In the absence of direct evidence of host–parasite relationships, we discuss the possible hosts of these Microsporidia, which could be the flatworms themselves or their prey; the most likely hypothesis is that they are parasites of land flatworms. Other *Mitosporidium* species should be sought for in native land flatworms from the Australasian region, where the two invasive flatworm species originated. Investigations on the possible pathogenic role of these parasites are needed.

## Abstract (fr)

Selon l’hypothèse classique du « relâchement de la pression des ennemis », l’une des raisons du succès des espèces envahissantes est qu’elles ont échappé à leurs prédateurs et leurs parasites lors de leur migration vers les territoires nouvellement envahis. Dans ce contexte, la découverte de tout parasite d’une espèce envahissante revêt un intérêt particulier. Nous présentons ici les résultats d’études génomiques réalisées sur deux espèces envahissantes de vers plats terrestres (Geoplanidae) collectées en Irlande du Nord : *Kontikia andersoni* Jones (1981) et *Australoplana sanguinea* (Moseley (1877). Nous décrivons les mitogénomes et les gènes d’ARN paralogues des deux espèces. De l’ADN de proie a été détecté chez les deux espèces de vers plats, apportant une preuve moléculaire que leur régime alimentaire comprend des vers de terre. De manière inattendue, nous avons détecté des séquences attribuées au genre de Microsporidies *Mitosporidium* Haag *et al.*, 2015, qui, avant cette étude, ne comprenait qu’une seule espèce. Chaque espèce de ver plat terrestre hébergeait sa propre espèce de *Mitosporidium*. Pour des raisons de nomenclature, nous n’avons pas pu attribuer de noms binomiaux à ces espèces; nous les désignons plutôt comme *Mitosporidium* sp. JL467 (chez *K. andersoni*) et *Mitosporidium* sp. JL472 (chez *A. sanguinea*). Pour chaque nouvelle espèce de *Mitosporidium*, nous décrivons le contenu génétique du mitogénome et le groupe complet des gènes de l’ARN ribosomique nucléaire. En l’absence de preuve directe de relations hôte-parasite, nous discutons des hôtes possibles de ces Microsporidies, qui pourraient être les vers plats eux-mêmes ou leurs proies; l’hypothèse la plus probable est qu’ils sont des parasites de vers plats terrestres. D’autres espèces de *Mitosporidium* devraient être recherchées chez les vers plats terrestres indigènes de la région australasienne, d’où sont originaires les deux espèces de vers plats. Des études sur le rôle pathogène de ces parasites sont nécessaires.

## Introduction

In continental Europe, the invasion by more than a dozen species of terrestrial flatworms has been an increasing cause for concern in recent years [2, 12, 13, 18, 25, 33, 34, 55, 56, 59–62, 70, 76, 78, 90, 91, 94, 96]. Very recently (July 2025), three species of geoplanids were added to the list of invasive alien species of Union concern [32].

In the British Isles, the most widely established invasive flatworm species is the New Zealand flatworm, *Arthurdendyus triangulatus* (Dendy, 1894) Jones & Gerard, 1999, whose spread, impact and pest management options have been scrutinized since the 1970s [1, 8–11, 16, 21, 48, 75, 77]. *Arthurdendyus triangulatus* was added to the list of species of Union Concern in 2019 [31]. For this species, our team has recently described the complete and complex mitochondrial genome and its paralogous clusters of nuclear rRNAs [39].

For several years, genomic studies on Geoplanidae have been developed, revealing original features of these animals [36–40, 42, 43, 54, 55, 57, 58, 88, 89]. As a part of these investigations, we collected additional species in Northern Ireland. The first species was the indigenous *Microplana scharffi* (Graff, 1899), from the subfamily Microplaninae Pantin, 1953, for which we described the mitogenome and additional genomic features [40]. Specimens of *Kontikia andersoni* Jones, 1981 and *Australoplana sanguinea* (Moseley, 1877) were also collected. The aim of our study was initially to populate the databases with sequences of poorly investigated organisms. The scarcity of molecular studies was exemplified by *K. andersoni*, for which not a single sequence was available on GenBank prior to this study.

While processing the sequencing results, it appeared that both species showed molecular signals of the presence of two species of the genus *Mitosporidium* Haag, James, Pombert, Larsson, Schaer, Refardt & Ebert, 2015 [46, 47]. Ten years after its description, the genus *Mitosporidium* still only includes a single species, *Mitosporidium daphniae* Haag *et al.*, 2015 [46, 47], an intracellular parasite of the guts of the planktonic crustacean *Daphnia magna* Straus, 1820 from northwestern Europe (Belgium, Germany, and the United Kingdom). *Mitosporidium daphniae* shares similarities with Microsporidia (*e.g.* their status as intracellular parasites, and the presence of a polar tube in spores), but it is clearly distinguished on the evolutionary level by having retained a restricted mitochondrial genome that contains a limited set of protein-coding genes (*vs.* no mitochondrion in Microsporidia). The circularity of this mitogenome remains to be verified.

The phylogenetic classification of *Mitosporidium* is not a matter of consensus, within the framework of the relationships between “true” Microsporidia and Rozellomycota and other basal Fungi [45]. *Mitosporidium* is either considered to be external to Microsporidia [30] or a member of “expended” Microsporidia [7], and sometimes designated as “short-branch microsporidian” [27]. In the most recent (2024) classification of Fungi, *Mitosporidium* is considered to be a member of the Phylum Rozellomycota, although its more precise placement is not provided [99]. This discussion is out of the scope of this paper; here we follow the interpretation of *Mitosporidium* as a member of the Microsporidia.

This serendipitous discovery of two species of *Mitosporidium* in invasive flatworms and its implications are described here, alongside all the information gathered in the course of this study concerning the genomics of the flatworm hosts and their preys. We also discuss whether these *Mitosporidium* species are actually parasites of the flatworms.

## Materials and methods

### Sampling

**Figures 1–4:**
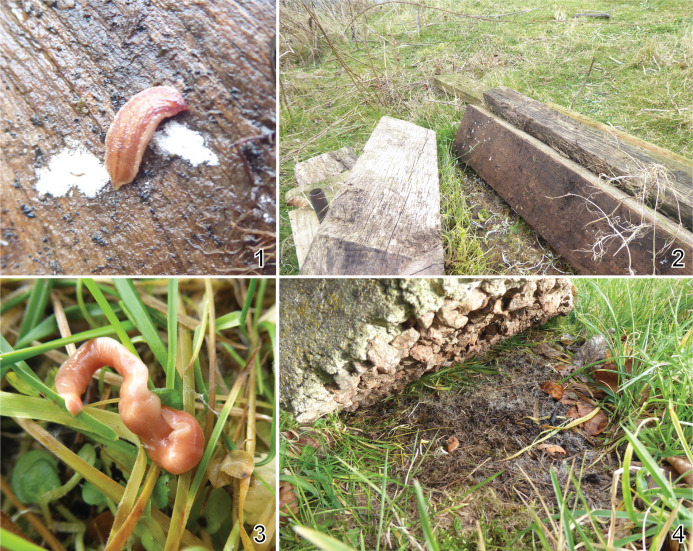
Geoplanids used in this study and their close environment. 1–2, *Kontikia andersoni*. 1, specimen MNHN JL467, unscaled; 2; origin of the specimen, below decaying wooden boards. 3–4, *Australoplana sanguinea*. 3, specimen MNHN JL472, unscaled; 4, origin of the specimen, below a concrete stone. Photographs by Stewart Rosell.

Flatworm collection took place in 2023. A specimen of *K. andersoni* was found by Mr Stewart Rosell (AFBI PhD student) in Castle Espie (latitude 54.53152, longitude -5.69632), a wetland reserve located in County Down, Northern Ireland, near a freshwater lagoon and below a decaying wooden board (Figs. 1–2). A specimen of *Australoplana sanguinea* was found in a private urban garden (latitude 54.588119, longitude -5.90957) in the main city of Belfast, also in County Down, below a concrete stone (Figs. 3–4) and adjacent to a compost box. Both specimens were killed by immersion in ethanol 96%, and kept stored within the ethanol before being sent to the National Museum of Natural History, France where they were registered in the collections under accession numbers MNHN JL467 for *K. andersoni* and MNHN JL472 for *A. sanguinea*.

### Genomics and bioinformatics

Due to its small size, the complete specimen of *K. andersoni* was used for sequencing, while just a posterior piece of 1 cm of *A. sanguinea* was cut and used. Samples were sent to the Beijing Genomics Institute to be sequenced on a DNBSEQ-G400 platform. Both samples returned a final quantity of *ca.* 140 M of 100 bp clean paired-end reads. Reads were analysed with Kraken 2 [102], version 2.1.3 against the NCBI core_nt database (k2_core_nt_20250609 release) with default parameters, using the THOT superdome flex server of the Laval University (Québec, Canada). Reads were assembled using SPAdes 4.0 [5] with k-mer 85, and the resulting contigs files were also analysed with Kraken 2. The contigs files were later datamined for the genes or genomes of interest by customised standalone blastn queries [15] using the mitogenome (OR835203) and the rRNA clusters (OR797296; OR797297) of *A. triangulatus* as references. Prey DNA was datamined as explained in previous publications [55]. Protein-coding genes of all mitochondrial genomes, whether of geoplanids or *Mitosporidium* spp., were annotated with the help of MITOS [28] with manual verifications done by blatsp queries of the open-reading frames displayed by ORFfinder (https://www.ncbi.nlm.nih.gov/orffinder/). Mitochondrial tRNA were found using ARWEN, v1.2 [67], while mitochondrial rRNA genes were found by manual alignment of the sequences with reference sequences from *A. triangulatus* using MEGAX [66], except for the nuclear rRNA genes whose boundaries were found with the help of Rfam [79]. LOGO representation of the amino-acid alignments were done on the LOGO website (https://weblogo.threeplusone.com/).

All data are available (see Data availability statement).

### Phylogenies

For geoplanids, a recent multiprotein dataset [41] established with the amino-acid sequences of the 12 proteins was appended with the sequences of *K. andersoni* and *A. sanguinea*. Since, as explained later, *ND4L* could not be found for *K. andersoni*, it was replaced by a blank sequence in the dataset. For *Mitosporidium* spp., the mitochondrial protein phylogeny dataset from Haag *et al.* (2014) [46], which consists of ATP6, Cob, Cox1, Cox2 and Cox3, was appended with the corresponding microsporidian sequences detected in the flatworms. Corresponding sequences from *Paramicrosporidium saccamoebae* Corsaro *et al.*, 2014 (GenBank: CM008827), a parasite of Amoeba, also considered a basal microsporidian [22], were also added. All proteins were independently aligned using MAFFT 7 [63] with the -auto option and the resulting alignment trimmed using trimAl [17] with the -automated1 and -keepseqs option. The best model of evolution was obtained on each alignment using ModelTest-NG [23] (default option). The alignments were then concatenated with Phyutility 2.7.1 [86]. All phylogenies were conducted using IQ-TREE 2.2.0 [72] with 1 000 ultrafast bootstrap replicates, and with a dataset partitioned with respect to the best model of evolution found for each alignment. All alignments and partition files are available as explained in the Data availability statement.

### Attempts to directly observe flatworm tissues to detect microsporidia spores

A small piece of specimen MNHN JL472 of *A. sanguinea* kept in ethanol was rehydrated in water and squashed between a slide and a cover glass. The slide was observed (by JLJ) with a differential interference contrast microscope at various magnifications for seeking spores. Since the whole specimen of *K. andersoni* was destroyed for molecular analysis, no such study was attempted. Stained sections of four specimens of *A. sanguinea* in the personal collection of one of us (LW) were re-examined, looking for microsporidia: MUZD 555 from Victoria H&E (Heidenhain’s Iron Haematoxylin); LW 890 from Tasmania (H&E, MSB trichrome); LW 1761 from South Australia (H&E, MSB trichrome), and LW 1806 A and B from Studley Hill, Lancs UK, *via* Hugh Jones (Trichrome).

## Results

### Taxonomic distribution of the reads and contigs

The vast majority of reads could not be classified by Kraken 2, with 88.39% and 83.87% of unclassified reads for *K. andersoni* and *A. sanguinea*, respectively. Only 1.45% and 0.9% of reads were assigned to Platyhelminthes. However, it should be noted that no reference nuclear genome is currently available for geoplanids in GenBank, which could explain the low assignment rates. The proportions of reads assigned to annelids were 0.24% and 0.23%, to arthropods 2.11% and 3.39% and to *Mitosporidium* spp. 0.08% and 0.17%, for *K. andersoni* and *A. sanguinea*, respectively. The search for traces of other potential hosts or parasites was also inconclusive. Kraken assigned approximately 450 reads to Gregarinasina (considered 0.00% by Kraken) and only 0.01% of reads to Amoebozoa.

As was the case for the reads, the majority of the contigs could not be classified. For *K. andersoni*, only 35.30% were assigned, but this percentage reached 48.72% for *A. sanguinea*. Out of these contigs, 0.53% and 0.44% were assigned to Platyhelminthes, which is lower than the percentage of reads previously assigned to this group. The percentages of contigs assigned to annelids were 0.15%/0.19%, to Arthropoda 6.25%/10.19%, to *Mitosporidium* spp. 0.01%/0.08%, to Gregarinasina 0.00%/0.00% and to Amoebozoa 0.03%/0.05% (for *K. andersoni/A. sanguinea*, respectively). It should be noted that these results somewhat contradict those obtained following the datamining for prey DNA, as explained below, especially for the overrepresentation of contigs assigned to Arthropoda.

### Geoplanids: paralogous rRNA genes

Like in all Geoplanids in which this was investigated, the two species studied here have two paralogous copies of the nuclear rRNA cluster of genes. Their nomenclature will follow those recently introduced [39]. For *K. andersoni,* partial (427 bp) sequences of both copies of the *18S* gene could be obtained from the contigs file, and showed 95.32% identity with each other. The low coverage copy (LCC), or type I, had coverage of 133.58× (GenBank: PV468224) and the high coverage copy (HCC), or type II, had coverage of 305.58× (GenBank: PV468223). For *A. sanguinea,* a partial 1,026 bp sequence of type I was obtained with coverage of 41.84× (GenBank: PV468328). Only 714 bp could be retrieved for type II, with coverage of 604.57× (GenBank: PV468329). After trimming and alignment with Clustal omega, the fragments showed only 92.10% identity with each other.

### Geoplanids: mitogenomes and phylogeny

For *K. andersoni*, the complete mitogenome could not be assembled; it was recovered as several contigs that could not be merged. However, it was possible to extract both rRNA genes plus eleven of the protein coding genes from these contigs (Table 1). *ATP6* displayed an alternative TTG codon, and *ND1* started with GTG. *ND4*, *ND5* and *cob* were partial. *ND4* was partial in 5′, while *cob* and *ND5* were partial in 3′ ending. It was thus impossible to verify whether or not *ND5* has a premature stop codon, as generally observed among species of Southern Hemisphere Rhynchodeminae [39]. Since it was also not possible to find *ND4L*, it is impossible to assess whether or not there is overlap between *ND4L* and *ND4*, as also reported [39]. The putative protein encoded by *cox2* is 461 amino acids long.

**Table 1:** Genes from the mitochondrial genome of *Kontikia andersoni.*

Gene	Length (bp)	Completeness	GenBank
*ATP6*	675	Complete	PV540689
*cox1*	1 620	Complete	PV540691
*cox2*	1 386	Complete	PV540692
*cox3*	795	Complete	PV540693
*ND1*	894	Complete	PV540694
*ND2*	954	Complete	PV540695
*ND3*	354	Complete	PV540696
*ND4L*	Not found	–	–
*ND4*	1 186	Not complete	PV540697
*ND5*	1 314	Not complete	PV540698
*ND6*	498	Complete	PV540699
*Cob*	966	Not complete	PV540690

**Figure 5:**
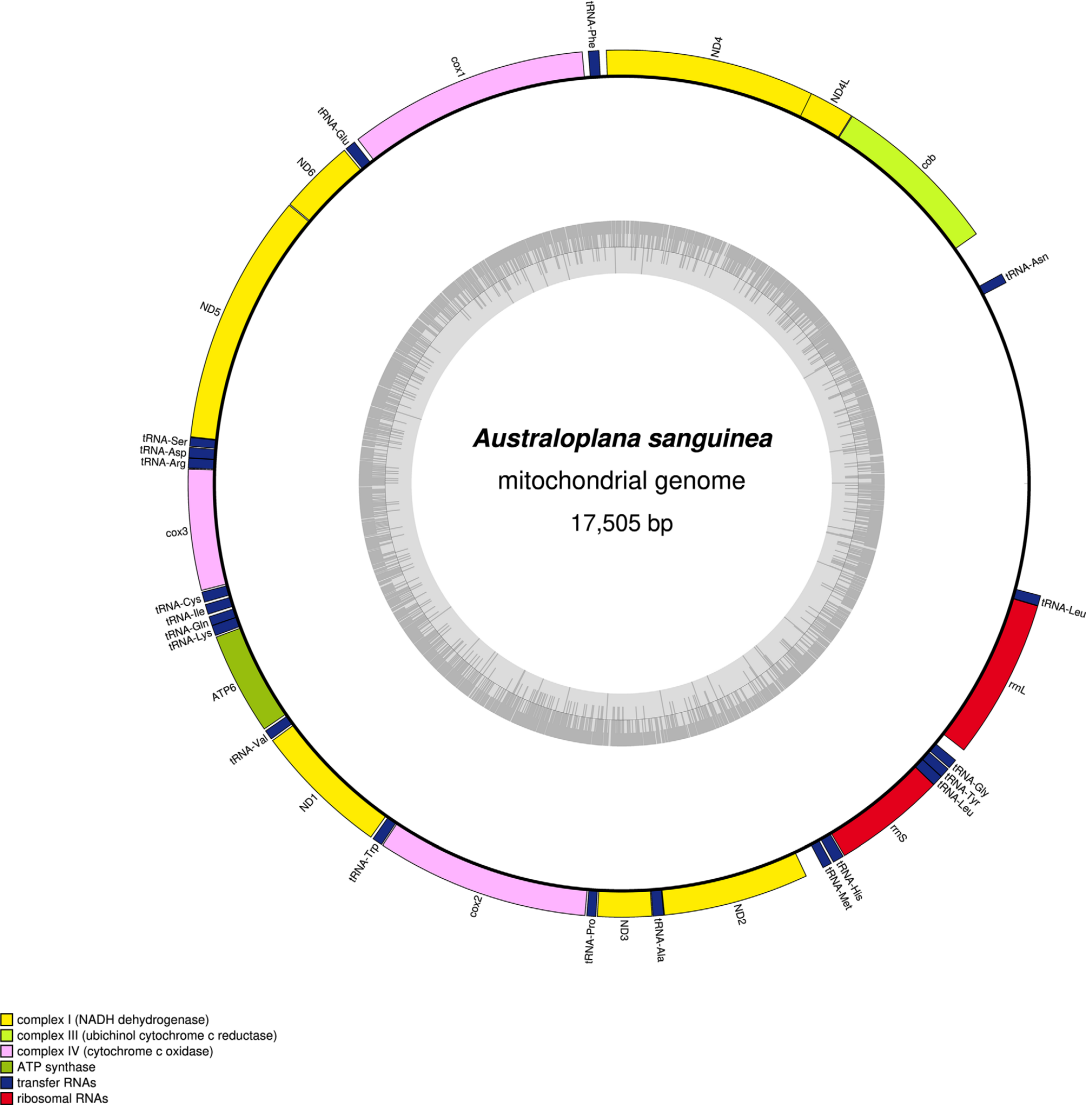
*Australoplana sanguinea*, mitogenome. The mitogenome is 17 505 bp in length and is represented as circular. The mitogenome codes for 12 protein coding genes, 20 tRNA and 2 rRNA.

For *A. sanguinea*, a 17 505 bp contig containing all genes was retrieved with 164.14× coverage. The genome could, however, not be circularized because of the lack of redundancy between its endings and the presence of repetitions, as it is known to occur among rhynchodemins [39]. For easier reading, it is represented as circular in Figure 5. The mitogenome codes for the 12 conserved protein coding genes, 20 tRNA and two rRNA (GenBank: PV491411). It was not possible to find *tRNA-Thr*, which is commonly lost or at least too divergent to be detected among geoplanids. Also, it was not possible to find one of the two *tRNA-Ser* usually found in rhynchodemins: more precisely, the *tRNA-Ser* that clusters with *tRNA-Leu*, *tRNA-Tyr* and *tRNA-Gly*, between the two rRNA genes. ARWEN suggested that a second *tRNA-Ser* might be present, but on the negative strand, and since all mitogenomes of geoplanids so far had their genes on the same strand, this result was not retained. The mitogenome shows several other features: *ND5* shows a premature stop because of the presence of *tRNA-Ser*; there is a 32-bp overlap between *ND4L* and *ND4*; *ND3* displays an alternative TTG start codon; and the putative Cox2 protein is 464 AA long.

**Figure 6:**
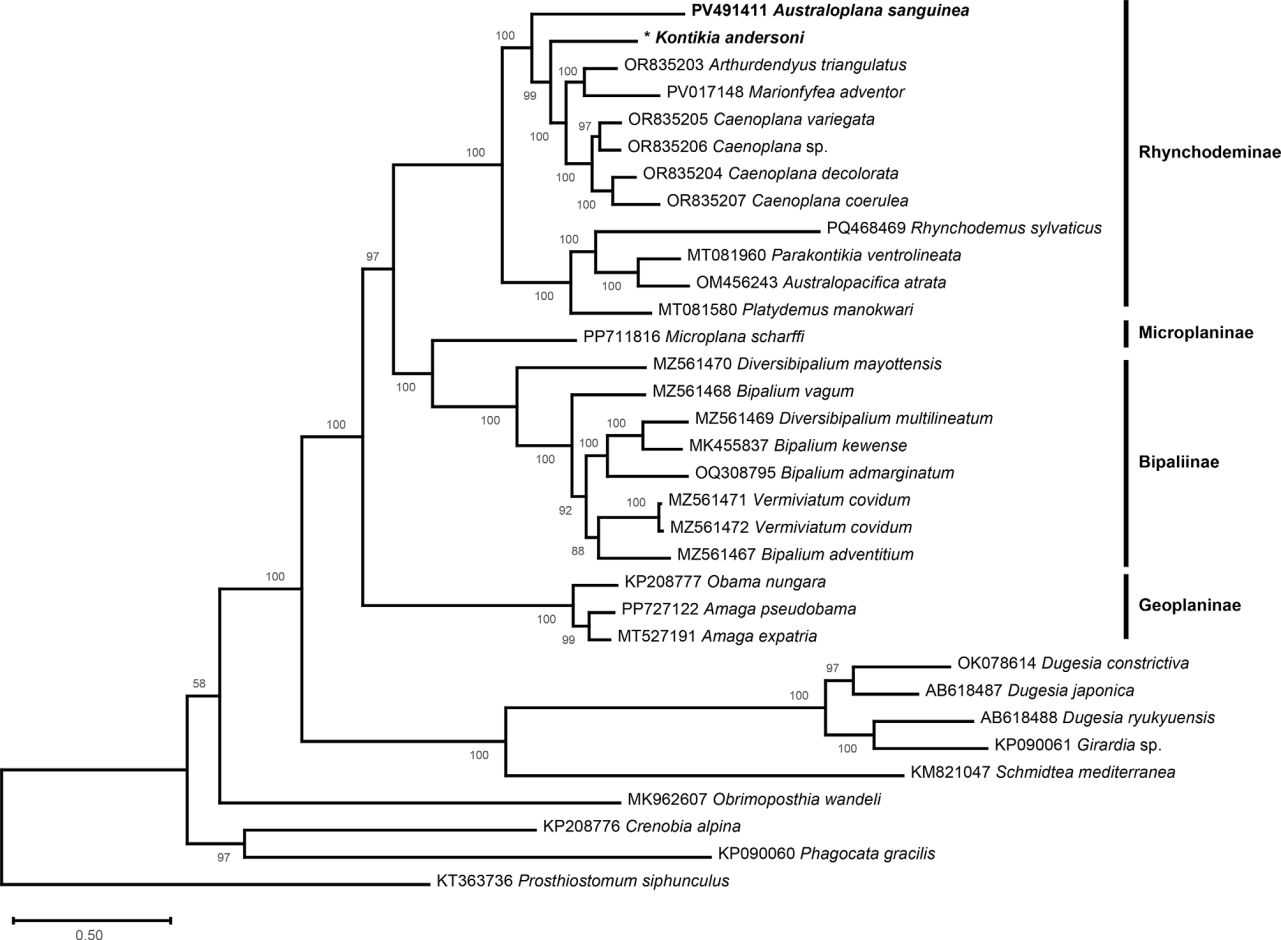
Maximum likelihood phylogenetic tree of geoplanids, based on concatenated amino acid sequences of 12 mitochondrial proteins. *For *Kontikia andersoni*, *ND4L*, which was not found, was replaced by a blank sequence in the dataset. Subfamilies are indicated on the right. Support indicated at the nodes.

In the multiprotein phylogeny, both taxa belong to a strongly supported clade (100% support) that also contains *A. triangulatus*, *Marionfyfea adventor* Jones & Sluys, 2016 and four species of *Caenoplana* Moseley, 1877 (Fig. 6).

## Detection of prey DNA

*Kontikia andersoni*. A 9 884 contig with coverage of 200.79× was found. Best megablast query returned the completely covered 3 405 bp long partial *28S* gene of *Lumbricus* sp. THS-2006 (DQ790041, [92]) with 99.79% identity. An attempt was made to again assemble the reads with a k-mer of 55. When datamining the resulting contigs file, it was possible to find a 1 043 bp contig with low coverage of 6.08× that returned 100% identity with partial 658 bp *cox1* sequences labelled as *Lumbricus festivus* (Savigny, 1826) (FJ937302, [84] and JN419206, [29]).

*Australoplana sanguinea*. A 2 674 bp long fragment with high coverage of 405.95× was retrieved. Megablast query returned *18S* hits for several species of earthworms, with the same percentage of identity of 99.94%. Names, authorities, accession numbers, lengths and references are indicated in Table S1 (Supplementary File 1). Also, a 15 822 bp contig with coverage of 60.42× was found, that matches with the mitogenome of a lumbricid. The contig has TAs at both ends, and contains all the conserved coding parts of a mitogenome. Best megablast result for the query of the *cox1* gene was a partial 658 bp *cox1* gene belonging to the lumbricid *Dendrobaena octaedra* (Savigny, 1826) (GenBank: JQ909014 [80]) with identity 96.48%, suggesting that a member of the genus *Dendrobaena* was a prey.

For none of our samples could any *18S* from an Arthropoda, Amoebozoa or Gregarinasina be found, although for Arthropoda, Kraken’s results suggested otherwise.

### Presence of *Mitosporidium*-like sequences

Four sequences, which we ascribe to *Mitosporidium* spp., were found among the contigs files for both samples, with pairs of corresponding sequences found within each file. These sequences correspond to two different species. The complete cluster of nuclear rRNA was retrieved from both samples. The cluster was 6 245 bp long for *K. andersoni* with 273.26× coverage (GenBank: PV480898) and 6 420 bp long for *A. sanguinea* with 73.01× coverage (GenBank: PV480899). In Table 2, the lengths of the different parts of the cluster and their conservation are compared for the two sequences discovered in this study. Megablast queries of the *18S* part returned only two results with similarity >90%. One belongs to the reference sequence from *Mitosporidium daphniae* (GenBank: MF278562) from Haag *et al.* (2014) [46], the other is ascribed to an uncultured and unidentified Cryptomycota from a water sample coming from a bog located in the state of Michigan, USA (GenBank: MZ923257), described in Quandt *et al.* (2023) [81].

**Table 2:** Lengths of the different parts of the cluster of nuclear rRNA and their conservation in the two sequences discovered in this study.

Specimen	*18S*	*ITS1*	*5.8S*	*ITS2*	*28S*
Mtspo_JL467 (from *K. andersoni*)	1828	316	155	401	3545
Mtspo_JL472 (from *A. sanguinea*)	1827	354	155	535	3549
*Mitosporidium daphniae*	1823	414	155	726	3724/3533*
Mtspo_JL467 *vs.* Mtspo_JL472	93.80%	48.64%	95.48%	53.15%	84.73%
Mtspo_JL467 *vs. Mitosporidium daphniae*	93.36%	49.04%	94.84%	52.64%	85.25%
Mtspo_JL472 *vs. Mitosporidium daphniae*	98.74%	52.71%	99.35%	57.09%	92.80%

* There were discrepancies between the length of the *28S* indicated by the authors and the length suggested by Rfam. The first value is the length as noted on GenBank; the second is the length measured in this study.

**Figure 7:**
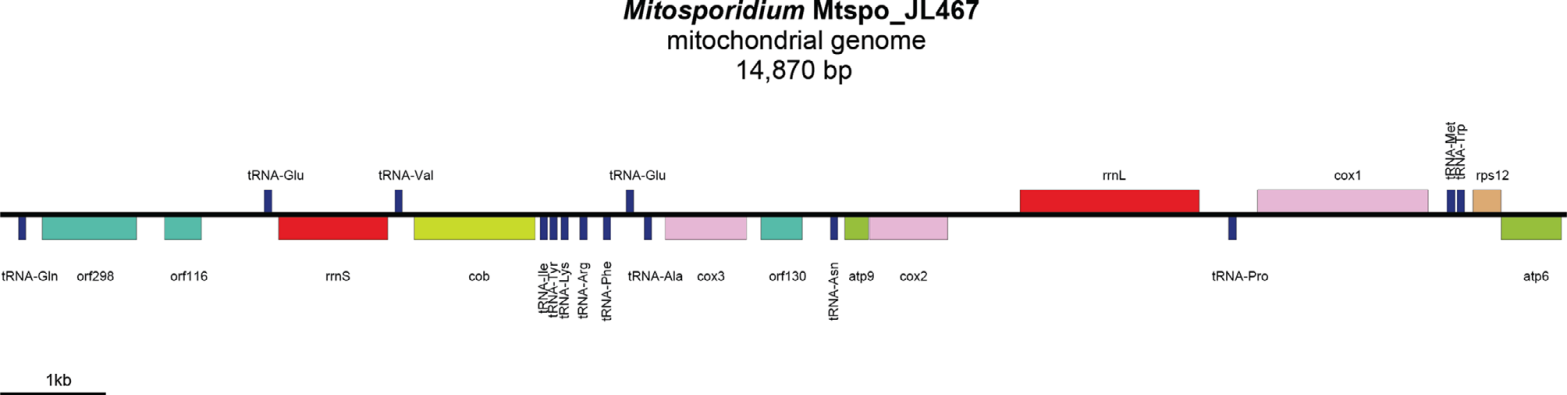
Mitogenome of *Mitosporidium* sp. JL467 (Mtspo_JL467) from *Kontikia andersoni*. The mitogenome is 14 870 bp in length. The mitogenome could not be circularised and is represented as linear.

**Figure 8:**
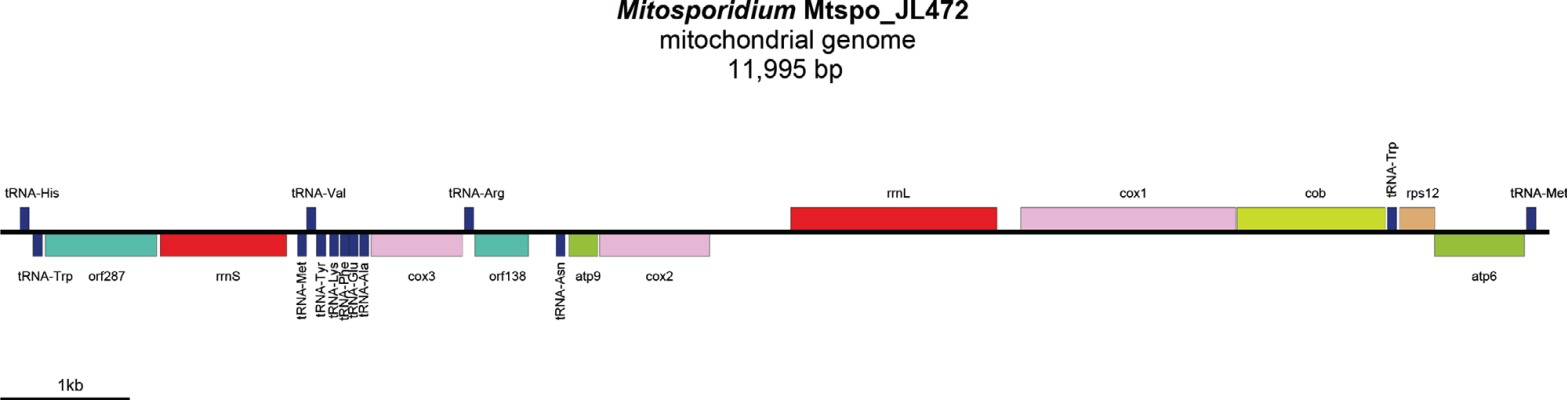
Mitogenome of *Mitosporidium* sp JL472 (Mtspo_JL472) from *Australoplana sanguinea*. The mitogenome is 11 985 bp in length. The mitogenome could not be circularised and is represented as linear.

The *Mitosporidium*-like mitogenomes will be referred to herein as Mtspo_JL467 (Fig. 7) and Mtspo_JL472 (Fig. 8), depending on the flatworm specimen they were found to be associated with. Their sizes were 14 870 bp (GenBank: PV491412) and 11 995 bp (GenBank: PV491413), with coverages of 26.33× and 34.89×, respectively. Their gene content was identical for the conserved protein-coding genes and rRNA genes, but differed regarding the non-conserved open-reading frames (ORFs) (Table 3) and the tRNA (Table 4). Except for the tRNA, the mitogenome of *Mitosporidium* sp. Mtspo_JL472 is colinear with that of *M. daphniae*, although it contains one less large non-conserved ORF. The mitogenome of *Mitosporidium* sp. Mtspo_JL472 shows a change in the position of the *cob* gene.

**Table 3:** Protein-coding genes, rRNA genes and non-conserved ORFs content of the mitochondrial genomes of *Mitosporidium daphniae*, *Mitosporidium* sp. Mtspo_JL467 and *Mitosporidium* sp. Mtspo_JL472.

Name	*Mitosporidium daphniae*	Mtspo_JL467 (from *Kontikia andersoni*)	Mtspo_JL472 (from *Australoplana sanguinea*)
Accession number	MW864067 [46]	PV491412 (this paper)	PV491413 (this paper)
complex I (NADH dehydrogenase)	–	–	–
complex III (ubiquinol cytochrome c reductase)	*cob*	*cob*	*cob*
complex IV (cytochrome c oxidase)	*cox1*, *cox2*, *cox3*	*cox1*, *cox2*, *cox3*	*cox1*, *cox2*, *cox3*
ATP synthase	*ATP6*, *ATP9*	*ATP6*, *ATP9*	*ATP6*, *ATP9*
SSU ribosomal proteins	*rps12*	*rps12*	*rps12*
LSU ribosomal proteins	–	–	–
rRNA	*rrnS*, *rrnL*	*rrnS*, *rrnL*	*rrnS*, *rrnL*
ORFs	orf138, orf292, orf295	orf116, orf130, orf298	orf138, orf287

**Table 4:** tRNA content of the mitochondrial genomes of *Mitosporidium daphniae*, *Mitosporidium* sp. Mtspo_JL467 and *Mitosporidium* sp. Mtspo_JL472.

tRNA	*Mitosporidium daphniae*	Mtspo_JL467 (from *Kontikia andersoni*)	Mtspo_JL472 (from *Australoplana sanguinea*)
*tRNA-Gly*	1	0	0
*tRNA-Pro*	1	1	0
*tRNA-Ala*	1	1	1
*tRNA-Val*	1	1	1
*tRNA-Leu*	1	0	0
*tRNA-Ile*	1	1	0
*tRNA-Met*	2	1	2
*tRNA-Cys*	0	0	0
*tRNA-Phe*	1	1	1
*tRNA-Tyr*	1	1	1
*tRNA-Trp*	0	1	2
*tRNA-His*	1	0	1
*tRNA-Lys*	1	1	1
*tRNA-Arg*	1	1	1
*tRNA-Gln*	0	1	0
*tRNA-Asn*	1	1	1
*tRNA-Glu*	1	2	1
*tRNA-Asp*	0	0	0
*tRNA-Ser*	1	0	0
*tRNA-Thr*	0	0	0

**Figure 9:**
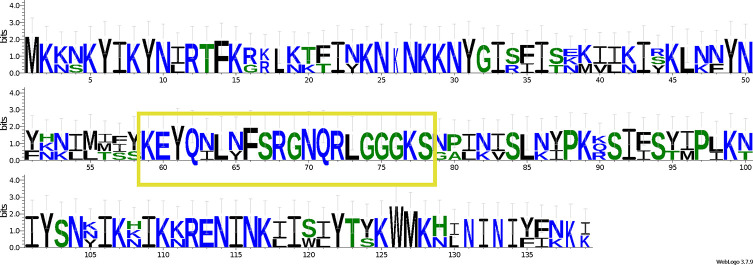
Alignment, presented as a LOGO figure, of the three putative proteins orf130 from *Mitosporidium* Mtspo_JL467, orf138 from *Mitosporidium* Mtspo_JL472 and orf138 from *Mitosporidium daphniae*. There is a nearly entirely conserved 20-amino-acid sequence in the central part (highlighted in yellow in the figure).

**Figure 10:**
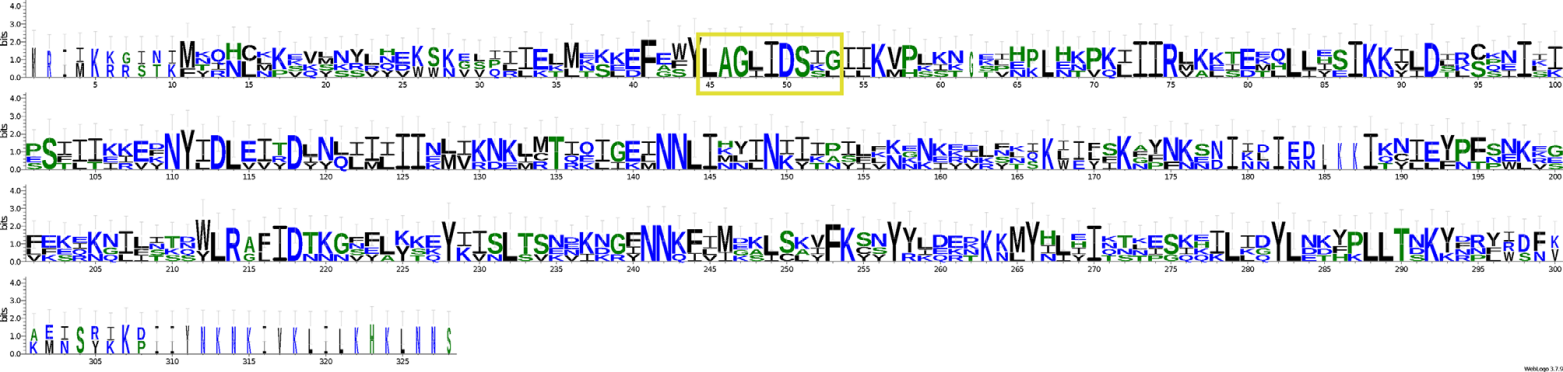
Alignment, presented as a LOGO figure, of the three putative proteins orf298 from *Mitosporidium* Mtspo_JL467, orf287 from *Mitosporidium* Mtspo_JL472 and orf295 from *Mitosporidium daphniae*. A partial LAGLIDADG domain was retained in the three species (highlighted in yellow in the figure).

Among the non-annotated ORFs, orf130 from Mtspo_JL467 and orf138 from Mtspo_JL472 were found to be well conserved with orf138 from *M. daphniae* (GenBank: QWQ66181). An alignment of these three putative proteins is provided as a LOGO figure (Fig. 9). In the central part, there is a nearly entirely conserved 20 amino-acid sequence that is highlighted on the figure. However, InterProScan could not find any noticeable conserved domain, and blastp queries just returned orf138 from *M. daphniae* as single result. This ORF is located between *ATP9* and *cox3* in the three *Mitosporidium* mitogenomes available. For orf116 from Mtspo_JL467, blastp queries suggest that it might be a truncated LAGLIDADG endonuclease. Blastp queries on orf298 from Mtspo_JL467 and orf287 from Mtspo_JL472 returned orf292 and orf295 from *M. daphniae* as best results. All seem to have retained at least partial LAGLIDADG domains, as can be seen in Figure 10.

There were noticeable differences in the content of tRNA between the three *Mitosporidium* mitogenomes. It should, however, be noted that *M. daphniae* was annotated using a different set of software, which could lead to discrepancies. All mitogenomes so far seem to lack *tRNA-Asp*, *tRNA-Thr* and *tRNA-Cys*.

**Figure 11:**
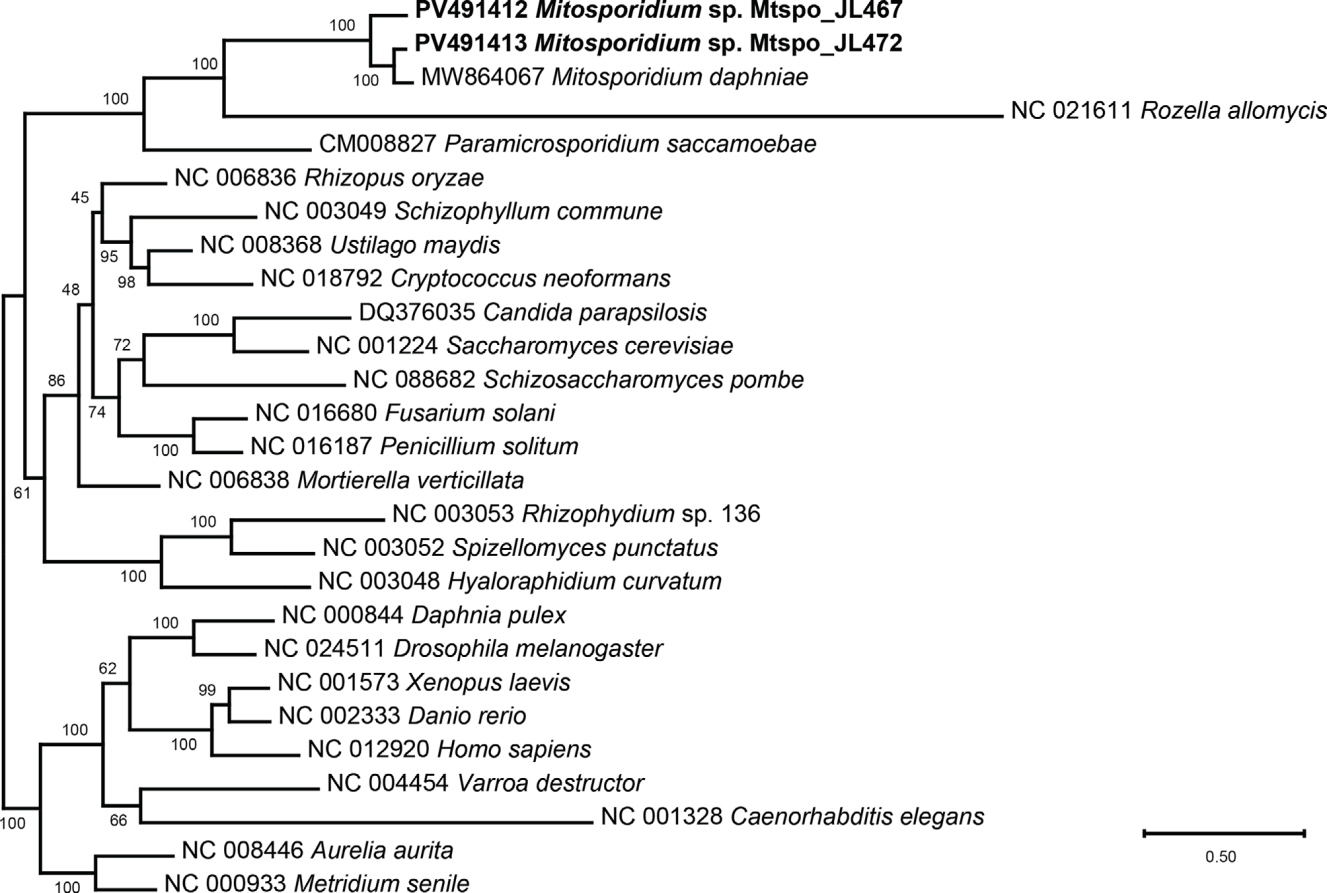
Maximum likelihood phylogenetic tree of the three *Mitosporidium* species and their relatives, based on concatenated amino acid sequences. Based on the dataset by Haag *et al.*, 2014 [46], added with the sequence of *Paramicrosporidium saccamoebae* and the sequences obtained in the present study for *Mitosporidium* Mtspo_JL467 and *Mitosporidium* Mtspo_JL472. The three *Mitosporidium* species are in a strongly supported clade, sister to *Rozella allomycetis*, which displays a very long branch, and *P. saccamoebae* is sister-group to the clade containing *Mitosporidium* and *Rozella.* Support indicated at the nodes.

The multiprotein phylogeny (Fig. 11) returned results similar to Haag *et al.* (2014) [46]. The three *Mitosporidium* species were in a strongly supported clade, sister to *Rozella allomycetis* (Doweld) Letcher*,* which displays a very long branch, and *Paramicrosporidium saccamoebae* was sister-group to the clade containing *Mitosporidium* and *Rozella. Mitosporidium daphniae* and *Mitosporidium* sp. Mtspo_JL472 were the closest related, which can be put into perspective with the complete conservation of the order of the protein-coding genes and rRNA genes among these two taxa.

### Search for microsporidia in flatworm tissues

We have not obtained direct evidence of microsporidia infection of flatworm tissue. No observation was made on *K. andersoni*. For *A. sanguinea*, our search for microsporidia on tissues kept in ethanol of the infected specimen MNHN JL472 was negative. Our search on stained serial sections of other specimens revealed evidence of gregarine infestation with the exception of the specimen from Tasmania. None exhibited anything in their intestinal diverticula that had the morphology of microsporidia, nor anything approaching Graff’s peculiar sporozoan [98].

### (In)formal description of two new species of *Mitosporidium*

Microsporidia are now known to belong to the Kingdom Fungi, but since they were considered unicellular protozoans in the past, the International Code of Zoological Nomenclature (ICZN) is still used for them. Therefore the rules of the current ICZN (1999) [50] apply to the description of new species. Two of these rules are the need for a description of the species (Article 13.1) and deposition of type-specimens (Article 16.4). We cannot satisfy the first of these rules, since our attempt to visualise spores in the *A. sanguinea* MNHN JL472 specimen failed, and the *K. andersoni* MNHN JL467 specimen was destroyed; for the second rule, we could consider that the remaining part of the body of specimen MNHN JL472 containing microsporidia is the type-bearing specimen, but this would be impossible for MNHN JL467, which was destroyed. Therefore, we present below what is currently known for each species, without ascribing a formal binomial name.

Both species are ascribed to the genus *Mitosporidium* Haag *et al.*, 2015 (Microsporidia) on the basis of high similarity of several DNA sequences (Tables 2–4).

Kingdom Fungi

Subkingdom Rozellomyceta

Genus *Mitosporidium* Haag, James, Pombert, Larsson, Schaer, Refardt & Ebert, 2015

(note that according to the ICZN, the date of the taxon is 2015, not 2014, since the 2014 paper did not comply with the Code) [46, 47].

### *Mitosporidium* sp. JL467

Locality: Castle Espie, Northern Ireland

Host: *Kontikia andersoni* Jones, 1981, Platyhelminthes, Tricladida, Geoplanidae

Possible hosts: species of *Lumbricus*, earthworm preys of the flatworm (see discussion)

Morphology: unknown

DNA characterisation: see Tables 2–4.

### *Mitosporidium* sp. JL472

Locality: Belfast, Northern Ireland

Host: *Austroplana sanguinea* (Moseley, 1877), Platyhelminthes, Tricladida, Geoplanidae

Possible hosts: species of *Lumbricus* (Supplemental Table S1) or species of *Dendrobaena*, earthworm preys of the flatworm (see Discussion)

Morphology: unknown

DNA characterisation: see Tables 2–4.

## Discussion

### Insights into two invasive terrestrial flatworm species

*Kontikia andersoni* supposedly originates from New Zealand. In Europe, it has been recorded in Cornwall, the Isles of Scilly, the Isle of Man, Scotland, Northern Ireland, and the Republic of Ireland [53, 100]. Various reports suggest that *K. andersoni* feeds on a wide range of prey that includes arthropods (especially Collembola), molluscs (slugs) and annelids (earthworms). The species has recently been introduced to the sub-Antarctic Macquarie Island [44, 101] where it is quickly spreading [49].

*Australoplana sanguinea* originates from South–East Australia, and is present in Tasmania, New Zealand and the Chatham Islands. Since its first record in the Isles of Scilly in 1980, it has colonized most of the British Isles including Ireland, Wales and Scotland and even the Channel Islands [3, 51, 52, 71, 85]. It seems particularly abundant in Cornwall and North–West England and is mostly known to feed on earthworms. For both species, no reports are known from continental Europe.

The original goal of this study was to document the genetic sequences of *K. andersoni* and *A. sanguinea*, for later use in molecular taxonomy and phylogeny. In this regard, and although the sequencing of *K. andersoni* was far from optimal, this task has been fulfilled. Genetic databases are now enriched with sequences of both species. It was possible in both cases to obtain partial sequences of both versions of the paralogous *18S* genes [19, 20, 39]. The multiprotein phylogeny clearly separates *K. andersoni* from *Parakontikia ventrolineata* (Dendy, 1892) Winsor, 1991 and *Australopacifica atrata* (Steel, 1897), two species that have previously been classified in the genus *Kontikia* Froehlich, 1955. This result strongly suggest that molecular phylogeny might challenge the current classification of several *Kontikia* species.

### Identification of prey

It is not possible to separate the organs when performing a molecular analysis on a fragment of the body of a flatworm, even more so when a whole specimen is used; for this reason, the sequences obtained include the flatworm itself, the contents of the digestive tract and therefore the prey, and (if present) the parasites, whether those of the flatworm or those of the prey.

For *Kontikia andersoni*, our molecular results based on both *28S* and *cox1* sequences suggest that the prey included species of *Lumbricus*, probably *L. festivus*. For *Austroplana sanguinea*, results based on a mitogenome suggest that the prey was a species of *Dendrobaena*, and results based on partial *18S* suggested that the prey included earthworms that could not be identified at the species level, which is expected for *18S* sequences [6]. This leaves us with the certainty that the prey of the two flatworms were earthworms.

No evidence of arthropod DNA (neither *18S* nor mitochondrial DNA) was found in the contigs file by data mining, although Kraken assigned between 2% and 3.4% of the reads to Arthropoda, and an even higher percentage of the contigs. Some of the largest contigs assigned to Arthropoda by Kraken were extracted and submitted to a Megablast query on the NCBI server. They did not return any results. Based on this, and on the discrepancies mentioned, we consider this to be primarily an artefact of the taxonomic assignment, which is probably impaired by the absence of a reference nuclear genome from a Geoplanidae in the database. In previous studies, our gut DNA datamining protocol [55] has successfully detected traces of multiple prey types within a single specimen. We must therefore conclude that no detectable amount of insect DNA was present in our samples, and that the Kraken assignments should be interpreted with caution. This point is particularly noteworthy, given that the only previously described species of *Mitosporidium* is a parasite of an arthropod.

### Considerations on *Mitosporidium* species

Based on sequence similarity across multiple loci, we have no doubt that the closest relative of our two species is *Mitosporidium daphniae*, and we therefore assign them to the genus *Mitosporidium.*

There were noticeable differences between Mtspo_JL467 and all related sequences, for both its nuclear rRNA or mitochondrial genome and despite the fact that the sample originates from the same area as Mtspo_JL472. This suggests that the radiation of *Mitosporidium* species includes major variations.

We would also like to note the conservation of the *ca.* 130**–**140 AA long ORF. Although it is impossible to assess whether or not this is a functional gene, in case more mitogenomes of *Mitosporidium* spp. became available, it would be worth checking the presence and conservation of this ORF.

### Hypotheses and speculations about the hosts of the two new species of Mitosporidium

On the basis of the available evidence, it is not currently possible to assess the exact host-parasite relationship between the two geoplanids investigated and the presence of *Mitosporidium* spp.

We briefly present here our reasoned doubts and speculations concerning host identity.

*Hypothesis 1*: The *Mitosporidium* spp. are parasites of flatworms. This is the simplest explanation, but we have not obtained direct evidence of microsporidia infection of flatworm tissue. This should be attempted by means of light and electron microscopy of various worm tissues. It is known that parasitic flatworms (Neodermata) can be parasitised by microsporidia [4, 68, 73, 87, 95]. However, no microsporidia appear to have been reported for terrestrial flatworms (Tricladida, Geoplanidae).

*Hypothesis 2*: The *Mitosporidium* spp. are parasites of earthworms, which are common prey for both flatworm species. Our results on the DNA of the prey indeed show that both flatworms consume earthworm species. However, only very few microsporidia parasitic in earthworms are known from an early light and electron microscopy study [14, 24], with none described with modern molecular methods.

If species of *Mitosporidium* are indeed parasites of earthworms and exhibit strict host specificity, then under Hypothesis 2, our findings could only be explained if two flatworm species, co-occurring in the same geographical area, each preyed upon a distinct earthworm species, and each of those earthworms was itself infected by a distinct *Mitosporidium* species. Such a scenario appears statistically far less plausible than Hypothesis 1, which posits that each *Mitosporidium* species is a parasite of a single flatworm species. In this latter case, our results would represent two independent flatworm–*Mitosporidium* host–parasite associations.

Both flatworm species studied here are native to the Australia–New Zealand region. This suggests the existence of radiation of *Mitosporidium* in the Geoplanidae of this region, which should be verified.

Alternative hypotheses may be proposed. The only species of *Mitosporidium* formerly described, *M. daphniae*, is a parasite of a freshwater crustacean. Neither geoplanids nor their earthworm preys have aquatic stages nor contact with freshwater animals; the hypothesis of a freshwater host is therefore rejected. The *Mitosporidium* detected here could be hyperparasites, *i.e.* parasites of parasites infecting flatworms or earthworms, such as amoebae. Indeed, some “basal” microsporidian species, such as *Paramicrosporidium saccamoebae*, parasitise amoebae [22]. However, given the absence of amoebal DNA in our samples, we dismiss this hypothesis.

The great similarities between the GenBank partial *18S* sequence MZ923257 [81] with *M. daphniae* and Mtspo_JL472 are puzzling. This sequence was derived from an aquatic sample [81]. If this sequence belongs to a species of *Mitosporidium*, What was its host?

## Conclusion

In this study, we characterised multiple sequences from two invasive flatworm species in Northern Ireland and, unexpectedly, detected the genetic signatures of two distinct microsporidian species – one in each flatworm host: *Mitosporidium* sp._JL472 in *Australoplana sanguinea* and *Mitosporidium* sp._JL467 in *Kontikia andersoni*. The two microsporidian species are genetically distinct yet both clearly belong to the genus *Mitosporidium*. Due to the limitations imposed by the ICZN, we are currently unable to formally describe these species or assign them Latin binomials.

As our study was based on a “cocktail” of DNA – including DNA of flatworms, earthworms and microsporidia – some uncertainty remains regarding the actual host of the microsporidia. We acknowledge that our conclusions rely on a single specimen per flatworm species, and that a definitive determination of host specificity will require additional sampling. Nevertheless, we consider the hypothesis that each *Mitosporidium* species is specific to its respective land flatworm host to be the more plausible explanation.

Following this study, the genus *Mitosporidium* comprises three species: one parasitic on a freshwater crustacean [46] and two parasitic on land flatworms. This significantly broadens the known host range of the genus.

A possible reason for the success of an invasive species when introduced into a new territory is the absence of predators and parasites, which allows the invasive species to reproduce exponentially; this is known as the Enemy Release Hypothesis [64]. Therefore, the discovery of parasites in invasive species is of particular interest. The case of the invasive yellow-legged (Asian) hornet (*Vespa velutina nigrithorax* Lepeletier, 1836) is emblematic [74], with the description of several parasites or pathogens [35, 65, 97], although none seem to reduce the spread of the species. *Mitosporidium daphniae* has been reported to exert a negative, albeit marginal, effect on the lifetime fecundity of its crustacean host [45, 83]. At present, nothing is known about the potential pathogenic effects of the newly identified *Mitosporidium* species on their respective hosts, and this question warrants further investigation.

## Data Availability

All data have been submitted to GenBank and have received accession numbers, as indicated in the text. In addition, the sequencing reads have been deposited in the SRA and are available under BioProject PRJNA1247450. Kraken reports, prey DNA, alignments and partition files are available on Zenodo: https://doi.org/10.5281/zenodo.17131052.

## Supplementary Files

*Supplementary Table 1:* *Australoplana sanguinea* Megablast query for a 2 674 bp long fragment with high coverage of 405.95×. The query returned *18S* hits for several species of earthworms, with the same percentage of identity of 99.94%. Names, authorities, accession numbers, lengths, reference and presence in Ireland are indicated. References include [26, 69, 82, 93]. The supplementary material of this article is available at https://www.parasite-journal.org/10.1051/parasite/2025060/olm.
