# Supplementary material for: An Irish cocktail of flatworm, earthworm and parasite DNAs: genomics of invasive land flatworms (Geoplanidae) reveal infestations by two new Mitosporidium species (Microsporidia)
Source: Parasite. 2025 Oct 17;32:67. doi: 10.1051/parasite/2025060 (PMC12534020; doi:10.1051/parasite/2025060)
Supplement: Supplementary file 1 — Supplementary Table 1: Australoplana sanguinea Megablast query for a 2 674 bp long fragment with high coverage of 405.95×. The query returned 18S hits for several species of earthworms, with the same percentage of identity of 99.94%. Names, authorities, accession numbers, lengths, reference and presence in Ireland are indicated. References include [26, 69, 82, 93]. [file parasite-32-67-s1.pdf]

Supplementary File

An Irish cocktail of flatworm, earthworm and parasite DNAs: genomics of invasive land flatworms (Geoplanidae) reveal infestations by two new *Mitosporidium* species (Microsporidia)

Romain Gastineau[1 *], Archie K. Murchie[2], Leigh Winsor[3], Jean-Lou Justine[4 *]

1. Institute of Marine and Environmental Sciences, University of Szczecin, Szczecin, Poland

2. Sustainable Agri-Food Sciences Division, Agri-Food and Biosciences Institute, Belfast, BT9 5PX, Northern Ireland, United Kingdom

3. College of Science and Engineering, James Cook University, Townsville, QLD, Australia

4. ISYEB, Institut de Systématique, Évolution, Biodiversité (UMR7205 CNRS, EPHE, MNHN, UPMC, Université des Antilles), Muséum National d'Histoire Naturelle, CP 51, 55 rue Buffon, 75231 Paris Cedex 05, France

Name	Authorities	Accession number	Length	Reference	Irish species
Aporrectodea trapezoides	(Dugès, 1828)	KF205934	1791 bp	Sun et al. (2017) [92]	
Aporrectodea caliginosa isolate E5	(Savigny, 1826)	PP534966	1772 bp	Raś et al. (2025) [82]	Yes
Aporrectodea caliginosa isolate E51	(Savigny, 1826)	PP534973	1761 bp	Raś et al. (2025) [82]	Yes
Aporrectodea caliginosa isolate E8	(Savigny, 1826)	PP534967	1749 bp	Raś et al. (2025) [82]	Yes
Dendrobaena platyura	(Fitzinger, 1833)	PP534969	1733 bp	Raś et al. (2025) [82]	No
Lumbricus rubellus	Hoffmeister, 1843	PP534975	1732 bp	Raś et al. (2025) [82]	Yes
Satchellius mammalis	(Savigny, 1826)	MW538114	1729 bp	Martinsson et al. (2021) [69]	Yes
Helodrilus oculatus	Hoffmeister, 1845	MW538113	1708 bp	Martinsson et al. (2021) [69]	Yes
Eiseniona gerardoi	Díaz Cosín, 2014	KF737140	1701 bp	Díaz Cosín et al. (2014) [26]	No

Supplementary Table 1: *Australoplana sanguinea* Megablast query for a 2,674 bp long fragment with a high coverage of 405.95×. The query returned 18S hits for several species of earthworms, with the same percentage of identity of 99.94%. Names, authorities, accession numbers, lengths, reference and presence in Ireland are indicated.

References

26. Díaz Cosín DJ, Novo M, Fernández R, Marchán DF, Gutiérrez M. 2014. A new earthworm species within a controversial genus: *Eiseniona gerardoi* sp. n. (Annelida, Lumbricidae)-description based on morphological and molecular data. ZooKeys, (399), 71.

69. Martinsson S, Klinth M, Erséus C. 2021. Distribution and genetic diversity of two earthworms, *Helodrilus oculatus* and *Satchellius mammalis* (Clitellata: Lumbricidae) in Scandinavia. Fauna Norvegica, 41, 1-14.

82. Raś D, Csuzdi C, Urbisz A, Gajda Ł, Małota K, Świątek P. 2025. Ovaries of Lumbricidae earthworms (Annelida, Crassiclitellata), from morphology to ultrastructure. European Zoological Journal, 92(1), 97-122.

92. Sun J, James SW, Jiang J, Yao B, Zhang L, Liu M, Qiu J, Hu F. 2017. Phylogenetic evaluation of *Amynthas* earthworms from South China reveals the initial ancestral state of spermathecae. Molecular Phylogenetics and Evolution, 115, 106-114.
